# The Single Application of External V‐Y Plasty in Correcting Alar Retraction

**DOI:** 10.1111/jocd.70242

**Published:** 2025-05-20

**Authors:** Li‐Yu Lai, Hsiu‐Cheng Hsu

**Affiliations:** ^1^ Department of General Medicine Changhua Christian Hospital Changhua Taiwan; ^2^ Department of Dermatology Changhua Christian Hospital Changhua Taiwan; ^3^ Department of Post‐Baccalaureate Medicine National Chung Hsing University Taichung Taiwan

**Keywords:** aesthetic effects, Asian patients, complications, dermatologic surgery, Mohs

## Abstract

With the rising incidence of basal cell carcinoma, postoperative deformities following Mohs surgery have become increasingly prevalent. Among these, alar retraction represents a particularly challenging complication. Current approaches to its correction have largely centered on grafting techniques, which are often complex and may necessitate multidisciplinary collaboration. External V‐Y plasty, by facilitating soft tissue lengthening and tension release, presents a straightforward and effective alternatives for correcting alar retraction, offering favorable aesthetic outcomes.

## 1 Introduction

Alar retraction is a troublesome issue often associated with inappropriate resection during rhinoplasty or excision of cutaneous malignancies. The deformity commonly refers to the proportionally uneven appearance between the alar and columellar relationship, as defined by Gunter et al. in 1996 [1]. Diverse grafting approaches have been developed to amend this disharmony; however, methods utilizing a purely cutaneous approach have been studied less extensively. Herein, the authors introduce a simple and effective method using a single external V‐Y plasty, which, to the best of our knowledge, has not been described in any previous articles.

## 2 Methods

**FIGURE 1:**
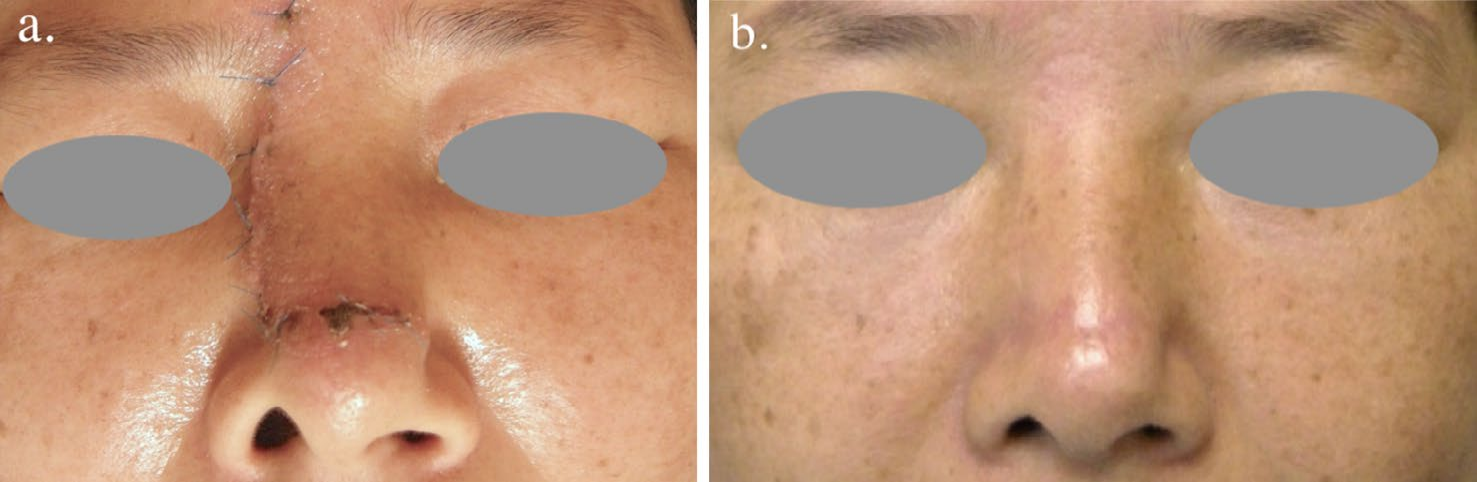
(a) Severe right alar retraction was noted as early as 2 weeks after the dorsal nasal flap procedure. (b) 6 months after correction with a single external V‐Y plasty, the outcome remained satisfactory.

This middle‐aged Asian woman presented with severe unilateral alar retraction deformity after receiving a dorsal nasal transposition flap performed for basal cell carcinoma excision (Figure 1a).

**FIGURE 2:**
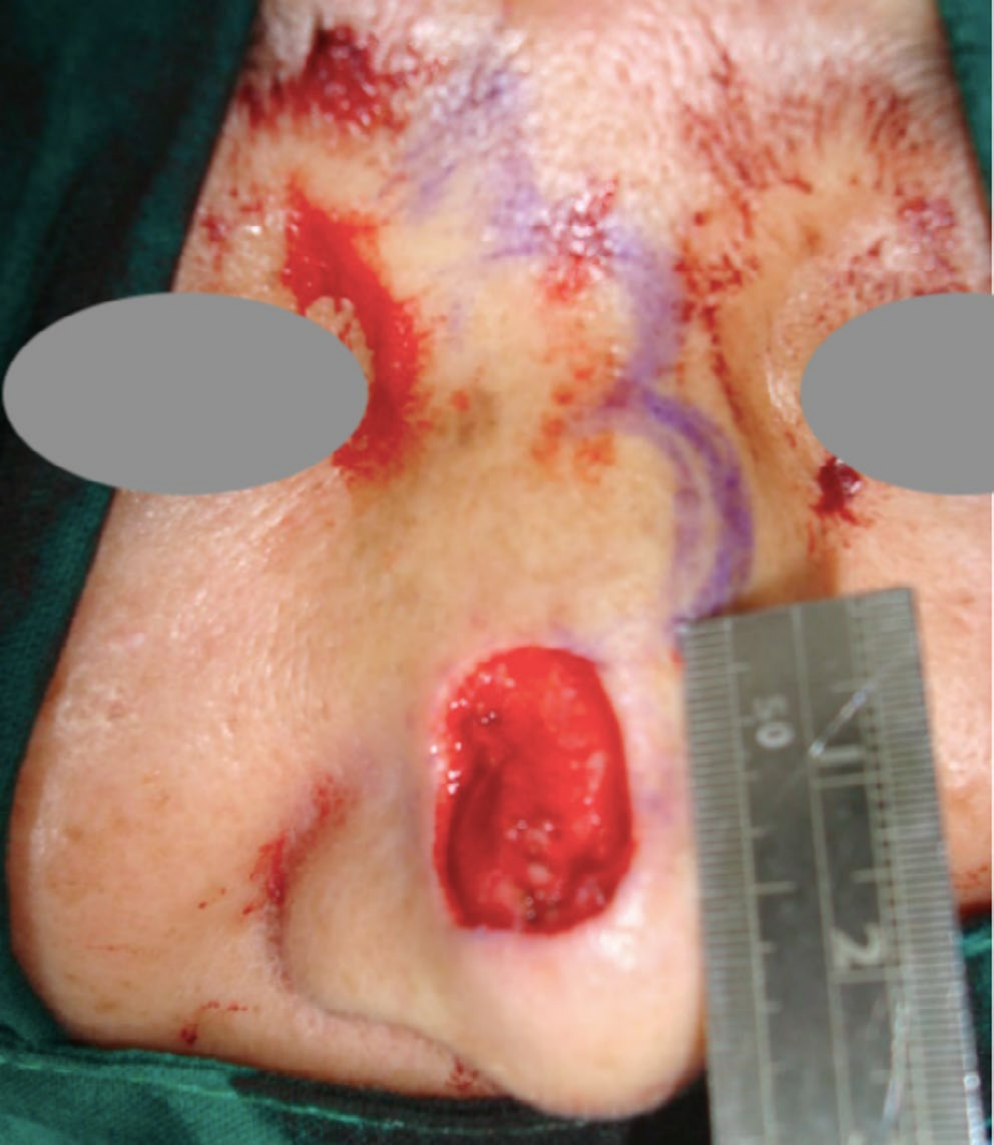
The original defect measured 1.8 × 1.5 cm following Mohs surgery. The remaining surgical marking reflected the initial plan for a trilobed flap, which was not adopted due to the patient's preference.

**FIGURE 3:**
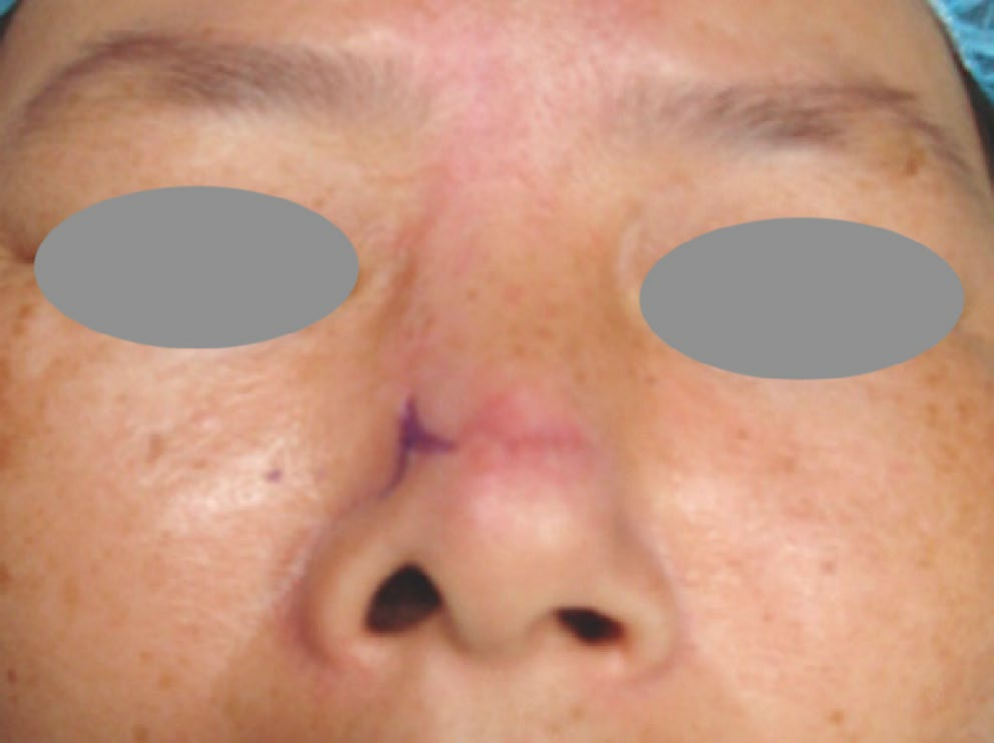
The V‐Y plasty was designed to be placed over the nasal sidewall.

The original defect measured 1.8 × 1.5 cm and was vertically oval after Mohs surgery (Figure 2), located over the supra‐nasal tip. A dorsal nasal flap was selected to conceal the incision in the lateral groove, in accordance with the patient's preference. The deformity was noted as early as 2 weeks postoperatively and showed minimal improvement over the following 3 months. There was no local necrosis, hypertrophic scarring, nasal cartilage involvement, or vestibular lining disruption during the original procedure. To address the deformity in a minimally invasive manner, a cutaneous V‐Y plasty was performed. The inverse V‐shaped incision was designed at the lower region of the sidewall, with the opening positioned near the supra‐alar crease (Figure 3).

## 3 Results

**FIGURE 4:**
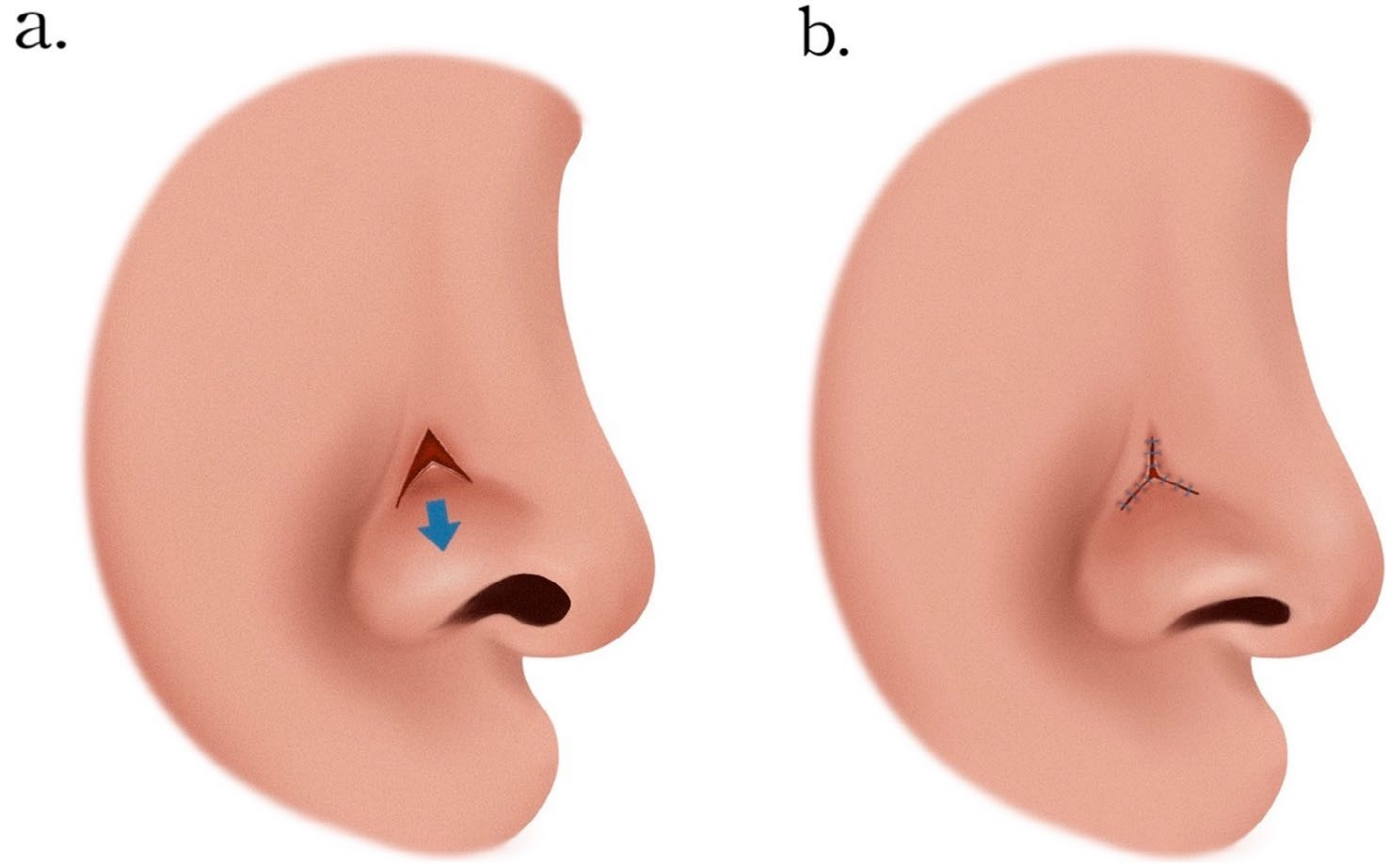
(a) An inverse V‐shaped incision was made at the lower sidewall near the supra‐alar crease, creating a triangular flap that was advanced caudally after full‐thickness dissection. (b) The natural alar rim contour was restored, and precise incision placement in the concave supra‐alar region could effectively minimize scarring.

By dissecting through the full thickness of the cutaneous layer, a triangular flap was created and regressed caudally (Figure 4a). This procedure effectively relieved the tension responsible for the upward pull of the skin, thereby restoring the natural contour of the alar rim (Figure 4b). The integrity of the cartilage and vestibular lining was preserved, and the correction was achieved without necessitating additional wounds elsewhere. At the 6‐month follow‐up, there was no recurrence of the deformity, nor were any additional complications observed. The patient expressed satisfaction with the improved symmetry of the alar region and the inconspicuous nature of the scar (Figure 1b).

## 4 Discussion

Recent research on the correction of alar retraction has mainly focused on cases arising secondary to rhinoplasty, in which resection of nasal cartilage and damage to the vestibular lining, along with cutaneous defects, are common contributing factors. Consequently, various approaches—such as composite grafts, lateral crural strut grafts, and alar grafts—are widely employed. A retrospective study of 45 patients with congenital or iatrogenic alar retraction, all treated by a single surgeon, illustrated the attribute of supportive grafts in achieving effective correction [2]. Specifically, these grafts were more likely to ensure proper alar rim positioning in patients with excessively narrowed lateral crura or cephalically oriented lower lateral cartilages [2]. Nevertheless, the utilization of tissue grafts, while enhancing stability of the alar rim, often results in more pronounced scarring.

To mitigate disparities in skin texture and color between donor and recipient sites, an island pedicle V‐Y flap, combined with an adjunctive composite graft, has been reported to successfully correct severe alar retraction in a deformity that developed 5 months after transposition flap reconstruction [3].

In the present case, the authors hypothesized that the primary cause of the alar retraction was closure tension and flap immobility, as the deformity developed shortly after the initial reconstruction. This differs from cases caused by scar contracture or over‐resection of cartilage. Thus, cutaneous approaches to release tension and restore a normal alar contour were preferentially considered. This method avoids the risk associated with pedicle flap necrosis and minimizes muscle destruction. The external V‐Y plasty was selected due to its nature to lengthen soft tissue, release tension, and its straightforward execution. Fine suturing and strategic placement of the incision at the concave supra‐alar region allowed for minimal scar formation. The decision to omit a cartilage graft was aimed at maximizing the cosmetic outcome, based on the assessment that there was minimal soft tissue loss over the alar.

The efficacy of a single external approach has been proposed by Yu et al. recently, who utilized Z‐plasty to ameliorate alar retraction in 23 Asian patients [4]. All patients, including 15 with an etiology of congenital anomalies, were satisfied with the correction during the 8‐month follow‐up [4]. Notably, each subject in both Yu's study and the present case was of Asian ethnicity. Given the limited research on using a single external flap alone to successfully correct alar retraction, this highlights the need for further investigation to establish patient selection criteria most suitable for this intervention.

## Consent

Patient consent of photography and publication has been retained by the authors.

## Conflicts of Interest

The authors declare no conflicts of interest.

## Data Availability

The data that support the findings of this study are available from the corresponding author upon reasonable request.
